# Assessing the social cost and benefits of a national requirement establishing antibiotic stewardship programs to prevent *Clostridioides difficile* infection in US hospitals

**DOI:** 10.1186/s13756-018-0459-1

**Published:** 2019-01-22

**Authors:** R. Douglas Scott, Rachel B. Slayton, Fernanda C. Lessa, James Baggs, Steven D. Culler, L. Clifford McDonald, John A. Jernigan

**Affiliations:** 1 Division of Healthcare Quality Promotion, National Center for Emerging and Zoonotic Diseases, Centers for Disease Control and Prevention (CDC), Roybal Campus, 1600 Clifton Road MS H16-3, Atlanta, GA 30329-4027 USA; 2 Division of Bacterial Diseases, National Center for Immunization and Respiratory Diseases, Centers for Disease Control and Prevention (CDC), Roybal Campus, 1600 Clifton Road MS-C25, Atlanta, GA 30329-4027 USA; 3 Department of Health Policy and Management, Rollins School of Public Health, Emory University, 1518 Clifton Road NE, Atlanta, GA 30322 USA

**Keywords:** Healthcare-associated infections, *Clostridioides difficile* infection, Antibiotic stewardship, Regulatory impact analysis, Value of statistical life, Cost-benefit analysis

## Abstract

**Backgound:**

Economic evaluations of interventions to prevent healthcare-associated infections in the United States rarely take the societal perspective and thus ignore the potential benefits of morbidity and mortality risk reductions. Using new Department of Health and Human Services guidelines for regulatory impact analysis, we developed a cost-benefit analyses of a national multifaceted, in-hospital *Clostridioides difficile* infection prevention program (including staffing an antibiotic stewardship program) that incorporated value of statistical life estimates to obtain economic values associated with morbidity and mortality risk reductions.

**Methods:**

We used a net present value model to assess costs and benefits associated with antibiotic stewardship programs. Model inputs included treatment costs, intervention costs, healthcare-associated *Clostridioides difficile* infection cases, attributable deaths, and the value of statistical life which was used to estimate the economic value of morbidity and mortality risk reductions.

**Results:**

From 2015 to 2020, total net benefits of the intervention to the healthcare system range from $300 million to $7.6 billion when values for morbidity and mortality risk reductions are ignored. Including these values, the net social benefits of the intervention range from $21 billion to $624 billion with the annualized net benefit of $25.5 billion under our most likely outcome scenario.

**Conclusions:**

Incorporating the economic value of morbidity and mortality risk reductions in economic evaluations of healthcare-associated infections will significantly increase the benefits resulting from prevention.

## Introduction

Healthcare-associated Infections (HAIs) pose a serious health threat to hospitalized patients with an estimated 4% of hospitalized patients in the United States (US) infected at any given time [1]. To mitigate this threat, actions are being taken by a myriad of public health organizations including government agencies, professional associations, private industry, and consumer groups. These actions include mandatory public reporting of hospital HAI rates and the formation of prevention collaboratives composed of multiple hospitals working together to prevent HAIs [2]. The US Congress passed several acts to empower agencies to implement polices to combat HAIs including the funding of states to develop HAI prevention plans, hospital reporting of select HAIs rates to the Centers for Medicare and Medicaid Services (CMS), and a penalty system to reduce hospital Medicare reimbursements for high rates of HAIs [3–5]. Additionally, the Executive Branch has launched efforts to coordinate various voluntary activities of both public and private sector stakeholders to achieve national goals of reducing HAIs and antibiotic resistant infections [6, 7].

As policy actions to combat HAIs and antibiotic drug resistance increase, economic evaluations of how to efficiently achieve national goals are needed to help inform policy decisions. However, conducting economic evaluations of policies affecting HAIs have been challenging given (1) the quality of hospital cost data, (2) the divergent cost perspectives (i.e. of patients, providers, third party payers, or society) that the analysis can take, (3) the methodological difficulties of assessing both short term and long-term attributable morbidities and mortality, and (4) difficulties in identifying patients with HAIs having their onset post-discharge [8–10]. Historically, most HAI economic analyses have taken the cost perspective of the healthcare provider and /or administrator [9, 11]. These studies attempt to demonstrate the cost savings to hospital budgets resulting from prevented cases of HAIs so as to make a ‘business case’ that investments in infection control reduce treatment costs and improve outcomes [12–14]. Using peer-reviewed attributable cost estimates for select HAIs, a 2012 meta-analysis found that the range of total direct medical costs to the US healthcare system due to hospital-onset central-line associated bloodstream infections, catheter-associated urinary tract infections, ventilator-associated pneumonia, surgical site infections and *Clostridioides difficile* infection (CDI) was $8.3–$11.5 billion (2012 dollars) [15]. While this work is still important, relying on analyses based on the provider cost perspective ignores the cost impacts to patients (travel costs, lost wages, long term morbidities, insurance co-pays), third party payers (increased per-patient reimbursements), and to society (mortality).

As required by executive order, US federal regulatory agencies conducting economic analyses of regulations impacting human health must take the societal perspective, which includes measuring the economic benefit of mortality risk reductions [16]. The US Office of Management and Budget (OMB) Circular A-4, Regulatory Analysis (2003) also directs regulatory agencies on use of the value of statistical Life (VSL) which is a monetized measure of the additional cost that individuals would be willing to pay for a small reduction in the risk of mortality [17]. An example from Robinson (2007) illustrates the VSL concept [18]. Assuming a population of 100,000, if each individual indicates they would be willing to pay an average of $50 to prevent one death (a risk reduction of 1/100,000), the VSL would be $50 X 100,000 or $5 million.

VSL estimates are derived from survey methods in which respondents are asked what they would be willing to pay for small changes in the risk of premature death (referred to as state-preference studies) or from statistical models that evaluate wage differentials for occupations with varying job-related mortality risks (referred to as revealed-preference studies). Concerns have been raised about the accuracy of VSL estimates from stated-preference studies, particularly related to the potential for hypothetical bias. As respondents are responding to a hypothetical market for risk reductions described within a questionnaire, respondents may provide “spurious” responses that do not reflect how they would respond to an actual market, and thus overstate (or understate) what they would actually pay for a reduction in risk [19–21]. However, there have been advances in both survey design and the use of statistical methods to make adjustments to responses if needed to minimize potential bias [20, 22]. Also, comparison of VSL estimates between revealed-preference and stated-preference studies have found that estimates from revealed-preference studies tend to be higher, which may be a reflection of how the perception of risk may differ between those who actually face the risk (as in wage studies), and those who may discount their own perceived individual risk to a hazard described in a survey [21]. While further research is needed to understand the discrepancies between VSL estimates from the two types of studies, certain types of mortality risk cannot be assessed using wage data (i.e. cancer) and require the use of stated-preference methods [20, 21].

Until recently, only the US Environmental Protection Agency (2014) and the US Department of Transportation (2015) had issued their own guidelines to ensure that their use of VSL in internal analyses complied with OMB directives [23, 24]. The US Department of Health and Human Services (HHS) has now published its own guidelines (2017) to inform and advise department agencies on the methods used in conducting regulatory impact analysis that are consistent with standing executive orders and OMB recommendations [25].

Prior to the HHS guideline publication, CMS had proposed a new rule to require that all hospitals certified by Medicare and/or Medicaid (4900 hospitals and 1300 critical access hospitals) establish and maintain antibiotic stewardship (AS) programs by 2020 as called for in the *National Action Plan for Combating Antibiotic-Resistant Bacteria* [7, 26]. While the rule also proposed other more modest requirements related to patients right to services (regardless of race, color or national origin), employment of an infection preventionist/infection control professional, mandated review of current infection control programs, and other nursing and medical record services, the requirement associated with the highest costs and benefits in the corresponding regulatory impact analysis was for AS programs. Using results from an economic evaluation of a multifaceted intervention (including AS programs) for CDI in US hospitals based on a Medicare cost perspective, the regulatory impact analysis concluded that requiring AS programs would result in annual savings of over 1 billion dollars to hospitals from reduced incidence of CDIs and drug cost savings from reduce inappropriate antibiotic prescribing [26, 27]. However, it was noted in the regulatory impact analysis that the potential societal benefits of reduced non-fatal CDI illness and the societal benefits and costs of reduced fatal CDI illness had been ignored due to lack of information on these benefits. A request for this information to be considered in the analysis of the finalized rule was made.

Our objective is to provide estimates of both the morbidity and mortality risk reductions associated with an active hospital AS program and enhanced infection control by expanding the previously mentioned Medicare analysis to incorporate a societal cost perspective. The VSL estimates, the methods for assessing intervention costs, and the methods for deriving the value of morbidity risk reductions from the VSL are taken from the new HHS guidelines. The possible ramifications of incorporating the societal perspective in evaluations of HAI prevention programs, and their interpretation by stakeholders and policy makers, will be discussed.

## Methods

This analysis relied on the results of two recent CDI studies to develop a net present value model to assess the social costs and benefits of a multifaceted CDI prevention program including the economic value of reduced mortality risks (see Appendix for model details) [27, 28]. Our economic model is partly based on a decision analytic (Markov) model developed by Slayton et al. to measure the net benefits of a multifaceted intervention (enhanced hospital infection control practices coupled with the implementation of an AS program) to prevent CDI in Medicare patients in acute care hospitals over a 5 year time horizon [27]. The simulation model incorporated information on projected hospitals discharges, infection incidence rates, intervention effectiveness, prevention program costs, and Medicare reimbursements saved per case averted, with each model representing a cohort of 1000 patients and outcomes assessed for 1000 trials. Using the cost perspective of the federal government (as a third party payer), the national financial savings to the Medicare program (under the base case scenario of 50% program effectiveness and a 3% discount rate) was estimated to be $2.5 billion (2011 dollars) with a credible range of $1.2 billion to $4.0 billion over the five year study period.

To expand this analysis from a Medicare cost perspective to reflect a societal cost perspective, we developed national estimates of the benefits of averted cases and reduced mortality risk for all ages using results from Lessa et al. which derived population-based estimates of the incidence and disease burden for (1) health care-associated *Clostridioides difficile* (HCA-CDI) (which included community-onset health care–associated, hospital-onset, and nursing home-onset infections) (2) recurrent HCA-CDI cases (within 14 to 56 days after the initial occurrence) stemming from these infections, and (3) the number of deaths occurring within 30 days after the diagnosis of HCA-CDI [28]. The net present value (NPV) model is defined as:

1 $$ \mathrm{NPV}={\sum}_{t=0}^5\frac{Benefits_t+{Costs}_t}{{\left(1+r\right)}^t} $$

where:

Benefits_t_ = the total benefits arising in year t (*t* = 0,1,2,3,4,5),

Costs_t_ = the total costs arising in year t (*t* = 0,1,2,3,4,5), and.

*r* = the social discount rate (3 and 7%).

The analysis of the net present value model was conducted using Excel for Windows 2016.

The study horizon for the measurement of costs and benefits in our societal analysis is a 6 year period beginning in 2015 through 2020 to be consistent with the published CMS analysis [26]. Unlike the Slayton analysis, we assumed a 3 month lag before benefits start to accrue in 2015 to account for the time needed to implement the intervention. Also, in contrast to the regulatory impact analysis done by CMS, we calculated the cost of the intervention to cover expenses to all inpatient prospective payment system (IPPS) hospitals as opposed to just 60%. The CMS analysis took into account that 40% of IPPS hospitals had already established ongoing AS programs, but we would argue that without the proposed requirement, hospitals would be free to suspend these programs given changes in any clinical or financial conditions facing the hospital [29]. To avoid any potential biases by limiting the coverage of the AS requirement, we fully assessed all cost and benefits that would accrue to all hospitals subject to the rule.

### Cost of prevention

The monetary unit costs of the CDI intervention program were taken from the Markov decision model and adjusted to 2015 dollars using the Consumer Price Index for Urban Consumers (CPI-U) (see Table 1) [27, 30]. Total intervention costs included the cost of implementing and staffing an AS program for HCA-CDI prevention (25% of total AS labor costs), the cost of implementing the Antimicrobial Use (AU) Option of the Antimicrobial Use and Resistance module of the National Healthcare Safety Network (the required data platform from CMS for assessing antibiotic prescribing), the federal government investment to hospitals to support adoption of AS programs, the cost to hospitals for patient isolation, hand hygiene, and enhanced environmental cleaning. As investments in AS programs and the AU module are broad-based interventions that can target other healthcare-associated organisms and reduce unnecessary drug use, only 25% of these costs are attributed to CDI prevention. To be consistent with the HHS guidelines, all labor costs that did not include overhead were doubled. Costs were expressed as costs per hospital discharge using 2015 discharges from National Inpatient Sample from the Healthcare Cost and Utilization Project as a base (35,232,942 discharges) and were combined with trend projections of the number of hospital discharges for 2015 through 2020 (see appendix for additional details) to estimate total prevention costs [31]. As recommended by the HHS guidelines, the analysis used discount rates of 3 and 7%.

**Table 1:** Net Economic Benefits Model for CDI Prevention: Model Inputs (2015 $)

Incidence Rates [28]	HCA-CDI rate per 100,000 persons	Recurrence Rate Per 100,000 persons	Death Rate Per 100,000 persons
Age Group			
1–17	6.3	0.4	NA
18–44	18.3	3.0	NA
45–64	83.1	10.9	5.4
≥ 65	481.5	117.6	55.1
Effectiveness of the Multifaceted CDI Intervention [27]	10 and 50%		
% of Total Deaths Due to CDI [28, 33]	35 and 50%		
Cost Inputs [27]	Cost Per Hospital Discharge		
Infection Control and Isolation Costs	$3.56 (50 effectiveness), –6.41 (10% effectiveness)		
Implementation of the Antimicrobial Use (AU) module			
Initial Cost (in 2015)	$0.08		
Ongoing Costs	$0.03		
Antibiotic Stewardship Personnel^a^			
(1.2 Pharmacists +0.67 Infectious Disease Physician + 0.05 Network Data Analysis) × 0.25	$20.58		
Federal Government Investment			
Initial Cost (2009–2014)	$0.18		
Ongoing Costs (2015)	$0.03		
Cost of Enhanced Cleaning	$0.28		
Total			
Initial cost (2015)			
50% program effectiveness	$24.68		
10% program effectiveness	$27.52		
On-going cost (2016–2020)			
50% program effectiveness	$24.47		
10% program effectiveness	$27.32		
Benefits of Prevention			
Attributable Patient Cost Savings			
HCA-CDI [27]	$ 6844 (Per Prevented Case)		
Recurrent CDI [27]	$12,703 (Per Prevented Case)		
Length Of Hospital Stay (LOS)^b^			
Mild/Moderate HCA-CDI Disease	9.5 days		
Recurrent Disease	8.8 days		
QALY/ adjusted to QALD by LOS^c^			
Mild/Moderate HCA-CDI Disease	0.80 / 0.005205479		
Recurrent Disease	0.708 / 0.00704		

*HCA-CDI* healthcare-associated *Clostridioides difficile* infection, *CDI Clostridioides difficile* infection*, QALY* quality-adjusted life year, *QALD* quality adjusted life day, *LOS* length of hospital stay, *AU* the Antimicrobial Use Option of the Antimicrobial Use and Resistance (AUR) module of the National Healthcare Safety Network (NHSN)

^a^Only 25% of total stewardship program and federal government investment costs were attributed to CDI prevention activities as these prevention efforts also involve other multi-drug resistant organisms

^b^Length of stay for mild/moderate HCA-CDI comes from Gabriel and Beriot-Mathiot while length of stay for recurrent CDI comes from McFarland et al. [37, 44]

^c^From Sullivan et al. the QALY weight selected for mild/moderate HCA-CDI disease is the 25th percentile EQ-5D index score which reflects a population that is older, with more comorbidities, and with a lower socio-demographic profile of respondents in the Medical Expenditure Panel Survey (MEPS) survey [42]. The QALY weight for recurrent HCA-CDI disease corresponds to the 25th percentile EQ-5D score for those older MEPS respondents with “Other Gastrointestinal Disorders” (clinical classification category 155). These weights are adjusted by the LOS associated with HCA-CDI and recurrent CDI disease to reflect the short term, acute nature of mild/moderate CDI disease (See the appendix for the adjustment formula to convert QALY to QALD).

The difference in the per-discharge cost of isolation and infection control ($3.56 versus $6.41) is the result of the different effectiveness levels and their impact on the number of HCA-CDI cases that will need enhanced infection control. At 50% program effectiveness, the number of cases that will need enhanced infection control is reduced by half, while at the 90% effectiveness level, the number of cases that will need enhanced infection control is only reduced by 10%.

### Benefits

Total benefits include the attributable patient treatment cost savings from averted HCA-CDI cases, an estimate of the reduce hospital expenditures (cost savings) on antibiotics due to AS oversite, and the value of morbidity and mortality risk reductions reflected in reduced cases and deaths due to the prevention of HCA-CDI. Using the age-stratified incidence rates from the Lessa study, estimates of the number of HCA-CDI, recurrent infections, and HCA-CDI associated deaths were made using 2014 US Census Bureau projections for the US population for 2015 through 2020, in each of the four age strata: 1–17, 18–44, 45–64, and 65 and over (Table 2) [32]. The estimates of the direct medical cost savings due to averted HCA-CDI and recurrent CDI cases were made by multiplying the averted cases by estimates of the attributable cost savings for hospital-onset and recurrent cases found in Kwon et al. (2012) [33]. While using the cost savings of averted hospital-onset cases as a cost surrogate for all HCA-CDI cases, the Kwon analysis showed that hospital-onset costs tend to be lower and thus serves as a conservative estimate of the cost savings from averted HCA-CDI cases.

**Table 2:** Model Inputs: Cases and Deaths Averted; VSL, Value Per QALY, Value per QALD 2015–2020 (2015 $)

	7% discount rate 10% effectiveness	3% discount rate 10% effectiveness	7% discount rate 50% effectiveness	3% discount rate, 50% effectiveness		
Cases Averted^a^						
Inpatient Cases Averted						
HCA-CDI	167,699	184,749	838,493	923,743		
Recurrent	35,892	39,555	179,460	197,775		
Total	203,591	224.304	1,017,953	1,121,518		
Deaths Averted						
35% Attributable Mortality	5625	6199	28,123	30,996		
50% Attributable Mortality	8035	8856	40,176	44,280		
VSL Estimates (2015 $)^b^						
3% DR	2015	2016	2017	2018	2019	2020
Low	$4,500,000	$4,368,932	$4,335,941	$4,301,166	$4,175,889	$4,140,522
Central	$9,400,000	$9,320,388	$9,143,180	$8,968,388	$8,884,870	$8,712,349
High	$14,400,000	$14,174,757	$13,950,419	$13,727,125	$13,505,003	$13,370,436
7% DR	2015	2016	2017	2018	2019	2020
Low	$4,500,000	$4,236,288	$3,959,148	$3,782,363	$3,611,765	$3,375,481
Central	$9,400,000	$9,037,415	$8,534,163	$8,140,304	$7,684,607	$7,253,694
High	$14,400,000	$13,744,402	$13,021,197	$12,333,794	$11,680,602	$11,060,088
Value Per QALY (2015 $)						
3% DR	2015	2016	2017	2018	2019	2020
Low	$222,833	$216,343	$214,709	$212,987	$206,784	$205,032
Central	$465,474	$461,532	$452,757	$444,101	$439,966	$431,423
High	$713,067	$701,913	$690,804	$679,747	$668,748	$662,084
7% DR	2015	2016	2017	2018	2019	2020
Low	$394,645	$377,023	$352,358	$336,466	$321,144	$300,135
Central	$833,140	$786,831	$750,676	$708,725	$669,051	$637,787
High	$1,271,634	$1,204,835	$1,141,334	$1,080,985	$1,023,648	$969,186
Value Per QALD (2015 $)						
3% DR - Cases	2015	2016	2017	2018	2019	2020
Low	$1160	$1126	$1118	$1109	$1076	$1067
Central	$2423	$2402	$2357	$2312	$2290	$2246
High	$3712	$3654	$3596	$3538	$3481	$3446
3% DR - Recurrent	2015	2016	2017	2018	2019	2020
Low	$1569	$1523	$1512	$1499	$1456	$1443
Central	$3277	$3249	$3187	$3126	$3097	$3037
High	$5020	$4941	$4863	$4785	$4708	$4661
7% DR - Cases	2015	2016	2017	2018	2019	2020
Low	$2054	$1963	$1834	$1751	$1672	$1562
Central	$4337	$4096	$3908	$3689	$3483	$3320
High	$6619	$6272	$5941	$5627	$5329	$5045
7% DR - Recurrent	2015	2016	2017	2018	2019	2020
Low	$2778	$2654	$2481	$2369	$2261	$2113
Central	$5865	$5539	$5285	$4989	$4710	$4490
High	$8952	$8482	$8035	$7610	$7206	$6823

*VSL* value of statistical life, *QALY* quality-adjusted life year, *QALD* quality-adjusted life day, *DR* discount rate.

^a^ To calculate the number of cases, we took national incidence rates from Lessa et al. (2015) and applied them to projections of the US population (by year of age) for 2015–2020 (United States Census Bureau 2014)) [24, 28]. Rates of attributable CDI mortality were derived by the authors’ based on analysis by Kwon et al. [33].

^b^The base estimates for the VSL were taken from the new HHS guidelines for conducting regulatory impact analysis (HHS 2017) [25].

For valuing mortality risk reductions, the HHS guidelines provide a range of VSL estimates to reflect the variability in these estimates from published studies and to promote the use of sensitivity analysis to assess the impact study on results. We used a low, a central and a high VSL estimate for the years 2015 to 2020, where we adjusted from 2014 VSL estimates to 2015 dollars using the CPI-U (Table 2) as recommended by the HHS guidelines. The guidelines also suggest that VSL estimates be adjusted to reflect changes in real income growth in future years (2015–2020), which we adjusted by 1.3% according to projections by the Congressional Budget Office [34]. Using the low, central and high VSL estimates to provide a range of benefits, each estimate was applied to every observed death regardless of age in accordance with the guidelines. The total economic value of the reductions in the risk of death due to the prevention program is estimated by multiplying the discounted estimate of the number of deaths averted in each year (2015–2020) by the corresponding discounted estimate of VSL and then summing across the years.

For this analysis, we estimated the value of morbidity risk reductions by assuming that the incidence of all cases (including recurrent cases) was mild or moderate. While cases of HCA-CDI can result in fulminate colitis (approximately 16%) and recurrent cases can experience up to 14 recurrent episodes, the source for our burden estimates did not categorize cases according to their disease severity or track when cases experienced multiple occurrences through time [28, 35–38]. Thus, our valuation of the morbidity risk reductions are conservative and should be considered a lower bound. As such, each case will only experience a decrease in utility once within each analytical year.

To value morbidity risk reductions, the HHS guidelines recommends the use of willingness-to-pay estimates that measure the maximum amount individuals would give up in income to pay for reducing the risk of illness. As no such estimates exists for HCA-CDI, the guidelines recommend the use of monetized quality-adjusted life years (QALYs) as a surrogate measure. To construct this value for morbidity risk reductions in the future, four pieces of information are needed: (1) the remaining years of life expectancy, (2) a health-related quality of life (HRQL) associated with each expected year of life that declines in quality of life due to age, (3) the probability of survival in each expected year of life, and (4) a monetary value per expected (monetarized) QALY (derived from the VSL). Items 1–3 are used to calculate expected QALYs associated with age by first multiplying the HRQL in each expected year of life by the probability of living in that year (i.e., by the survival curve) [39, 40]. The discounted sum of these expected QALYs are calculated over the remaining years of life expectancy using the same discount rates of 3 and 7%. For our study population, we assumed an average age of 40 with a life expectancy of 50 years. To get the monetarized QALY, the VSL estimates were then divided by the expected QALYs [41]. Our estimates for the monetary value per expected QALY for 2015–2020, based on the low, central and high VSL estimates, ranged from $223,000 to $1.27 billion (Table 2).

To derive an estimate of the monetary value of reducing the risk of getting a mild/moderate disease, HRQL weights associated with HCA-CDI and recurrent CDI disease are needed to adjust the monetary value per QALY. As there are no published HRQL weights specifically for HCA-CDI disease, we selected surrogate community preference-based EQ-5D index weights from Sullivan et al. (2006) [42]. For mild/moderate HCA-CDI cases, we selected the unadjusted 25th percentile EQ-5D score (taken from the Medical Expenditure Panel Survey 2000–2002) of 0.80 which can be interpreted as reflecting a patient population that is older with more comorbidities, which have been found to be risk factors for HCA- CDI disease [43]. For mild/moderate recurrent cases, we selected the unadjusted 25th percentile EQ-5D score for other gastrointestinal disorders (Chronic Classification Condition 155) of 0.708, which reflects an older patient profile with these diseases, to act as a surrogate for patients with recurrent CDI disease. The EQ-5D weights are adjusted to quality-adjusted life days (QALD) and then multiplied by the associated hospital lengths of stay for acute episodes of HCA-CDI and recurrent cases (9.5 and 8.8 days respectively from Table 1) to obtain an adjusted QALD lost from avoided HCA-CDI and recurrent infections (0.0052 and 0.007 respectively from Table 1 and Appendix) [37, 44]. These weights are then used to adjust the monetary value per QALY to get a monetary value per lost QALD from mild/moderate HCA-CDI and recurrent disease. To obtain the total value of morbidity risk reductions, the number of HCA-CDI and recurrent cases are then multiplied by their respective monetary value per lost QALD. Our estimates for the monetary value of QALD for mild/moderate HCA-CDI disease ranged from $1067 to $8952. (Table 2).

Another potential benefit from AS programs is the reduction in hospital expenditures for antibiotics as these programs reduce antibiotic prescribing through more appropriate use [45, 46]. While the Slayton study did not consider these benefits, the CMS analysis derived an annual estimate of $520 million (in 2003 dollars) in these savings based on information from a single study of a 124 bed hospital [47]. To obtain a more robust estimate of the expected reduced antibiotic expenditures by hospitals, we used a 2009 estimate of total antibiotic expenditures by US acute care and long-term care hospitals ($3.6 billion); adjusted it to 2015 dollars ($3.9 billion) using the CPI-U; and then multiplied this estimate by a representative percentage savings in annual antibiotic expenditures taken from published studies [26]. In reviewing studies of US hospitals, the cost reductions ranged from 10 to 37% [47–54]. For our model, we used a reduction in antibiotic costs of 20% which resulted in an annual estimated savings of $787 million in antibiotic expenditures. After adjusting for the 3-month lag in 2015, the total discounted value of these savings for 2015–2020 was $4.2 billion (3% discount rate) and $3.8 billion (7% discount rate).

### Sensitivity analysis

The model is evaluated using two intervention effectiveness scenarios of 50 and 10% (the same base and lower bound model values as used in Slayton) while assuming full program implementation costs for each level [27]. While the projected number of HCA-CDI associated cases and deaths were adjusted by 50 and 10% to reflect program effectiveness, associated deaths were further adjusted by 50 and 35% to provide a credible range of the attributable proportion of HCA-CDI deaths due to CDI disease that reflects the limitations of current methods used to attribute these outcomes [28, 33, 55]. Consistent with HHS guidance, our sensitivity analysis also includes calculations using a low ($4.5 million), central ($9.4 million) and high VSL estimate ($14.4 million) for 2015 (Table 2). As derived from the low, central and high estimates of the monetary value for a QALY, the monetary value of QALD used in value reduction of morbidity risks ranged from $1160 to $8952 to reflect the short duration of mild/moderate CDI disease. As recommended by the HHS guidelines, the analysis used discount rates of 3 and 7%.

As VSL estimates in the HHS guidelines are derived for a population between the ages of 18 to 65, the guidelines also recommend that when the affected population is very old, additional sensitivity analysis should be done using monetized QALY values. These values are then multiplied by the expected value of the number of life years gained (which will be smaller for older populations). As 62% of cases and 84% of deaths in the Lessa study occurred in patients 65 and over, we developed monetized QALY values using an age range of 65 to 90 years (as opposed to ages 40 to 90 years used to obtain the monetized QALYs for the morbidity risk values described above) to be used in place of both the VSL estimates and the previous estimates for the value of morbidity risk reductions. A description of how these alternative estimates were derived can be found in the Appendix. From Table 6, the monetarized QALYs for mortality risk reductions ranged from $342,948 to $1,578,597, while the monetized QALYs for morbidity risk reduction ranged from $1643 to $11,113.

## Results

In our high estimate scenario based on 50% program effectiveness and attributable mortality, the national intervention was projected to avert 1,017,953 total inpatient cases and prevent 40,176 deaths using a 7% discount rate, and 1,121,518 total inpatient cases and 44,280 deaths using a 3% discount rate over the study period (Table 2). Using the low estimates of program effectiveness and attributable mortality (10 and 35% respectively), the model projects that 203,591 total inpatient cases and 5625 deaths were averted at the 7% discount rate, and 224,304 total inpatient cases and 6199 deaths were averted at the 3% discount rate.

Also from Table 2, the projected cost of the prevention program ranged from $3.3 billion (7% discount rate) to $4.2 (3% discount rate) at 50% program effectiveness. At 10% program effectiveness, the program costs ranged from $3.7 billion (7% discount rate) to $4.7 billion (3% discount rate). The higher costs under the 10% program effectiveness scenario arise from the increased costs associated with implementing the enhanced infection control practices (to avoid transmission) around the remaining 90% of cases (as opposed to 50% of cases).

Without considering the value of morbidity and mortality risk reductions, the net benefits in reduced patient care costs and reduced antibiotic expenditures from the intervention ranged from $8.1 billion to $9.1 billion (subtracting direct medical cost savings of $13.3 billion and $11.4 billion from the intervention costs of $4.2 and $3.3 billion respectively) under the 50% intervention effectiveness scenarios, but these net benefits decreased in the 10% effectiveness scenarios to $1.3 billion (3% discount rate) to $1.6 billion (7% discount rate) (Table 3). However, when the values for morbidity and mortality risk reductions were included, net benefits from the intervention ranged from $24 billion to $626 billion across all scenarios (Table 3). The inclusion of the economic value of mortality risk reductions using VSL estimates overwhelmed the difference between the intervention costs and the benefits of both the direct medical cost savings and the value of morbidity risk reductions as the total net benefits are substantial. The proportion of the economic value of mortality risk reductions to total benefits ranged from 80% (lowest total benefit estimate) to 97% (highest total benefit estimate).

**Table 3:** Benefits and Costs of a Comprehensive CDI Prevention Program 2015–2020 (2015 $)

	7% discount rate, 10% effectiveness	3% discount rate, 10% effectiveness	7% discount rate, 50% effectiveness	3% discount rate, 50% effectiveness
Total Intervention Costs (in billions)	$3.7	$4.7	$3.3	$4.2
Total Benefits (in billions)				
Direct Medical Cost Savings				
Savings in Patient Care Costs	$1.5	$1.8	$7.6	$9.1
Savings in Hospital Expenditures for Antibiotics	$3.8	$4.2	$3.8	$4.2
Benefits of Morbidity Risk Reduction				
Low $/Lost QALY from HCA-CDI	$0.4	$0.3	$2.1	$1.3
Central $/ Lost QALY from HCA-CDI	$0.8	$0.6	$4.1	$2.8
High $/ Lost QALY from HCA-CDI	$1.3	$0.8	$6.3	$4.2
Benefits of Mortality Risk Reduction				
Low VSL				
35% Attributable Mortality Proportion	$22.1	$26.6	$111.2	$133.2
50% Attributable Mortality Proportion	$31.5	$38.0	$157.7	$190.2
Central VSL				
35% Attributable Mortality Proportion	$46.6	$56.2	$232.8	$280.8
50% Attributable Mortality Proportion	$66.5	$80.2	$332.6	$401.1
High VSL				
35% Attributable Mortality Proportion	$71.1	$85.7	$355.7	$428.7
50% Attributable Mortality Proportion	$102.7	$122.5	$507.8	$612.5
Range of Total Net Benefits				
Low	$24.1 - $33.5	$28.2 - $ 39.6	$121.4 - $167.9	$143.6 - $200.6
Central	$49.0 - $68.9	$58.1 - $ 82.1	$245.0 - $344.8	$292.7 - $413.0
High	$74.0 - $105.6	$87.8 - $124.6	$370.1 - $522.2	$442.0 - $625.8

*HCA-CDI* healthcare-associated *Clostridioides difficile* infection, *VSL* value of a statistical life, *QALY* quality-adjusted life year.

Even with VSL estimates that have been age adjusted to reflect the older age distribution of HCA-CDI patients, the intervention still produced a range of total net benefits of $3.7 billion to $14.5 billion under our lowest effectiveness scenario (10% program effectiveness, 35% attributable mortality proportion) (Table 7). As this same intervention program achieved an 80% reduction in hospital CDI cases in England from 2008 to 2012, the credible range of net benefits is $21.2 billion to $625.8 billion which are associated with the 50% program effectiveness scenarios (Tables 3 and 7) [56–58]. Given the evidence on program effectiveness and the theoretical and empirical uncertainties associated with age-adjusted VSL estimates, we suggest that a likely, but conservative, scenario outcome from a public policy perspective is $121.4 billion in total net benefits (50% program effectiveness, 3% discount rate, 35% attributable mortality proportion, and the low VSL estimate). This translates to an annualized net benefit of approximately $25.5 billion.

## Discussion

The societal cost perspective has rarely been considered in economic evaluations of HAI prevention programs, but doing so in accordance with HHS guidelines for conducting regulatory impact analysis may provide stakeholders and policy makers a broader view on the benefits of such programs. As our intervention costs ranged from $3.3 billion to $4.2 billion, these costs would have to be at least 28 times larger to overlap with our lowest benefit estimate of $121.4 under the 50% program effectiveness scenario (Table 3) and would have to quintuple to overlap with our lowest benefit estimate of $21.2 when using age-adjusted VSL estimates and the 50% program effectiveness scenario (Table 7).

While the benefits of reducing mortality risks comprised a vast majority of total net social benefits in our model, a number of other relevant benefits (cost savings) associated with CDI disease are ignored in this analysis. The most important of these include non-hospital medical costs (e.g., outpatient treatment and pharmacy costs), medical costs and the value of morbidity risks due to severe or long-term morbidities, lost labor productivity, and the economic impacts on family/caregivers. More broadly, AS programs also produce spillover benefits in terms of reducing rates of antibiotic resistance, although such economic impacts are diffuse and difficult to quantify and require more research to understand their full effects. However, the addition of these potential benefits just provides more support for the proposed rule. As the range of credible costs and benefits did not overlap, we did not perform a Monte Carlo simulation in our sensitivity analysis.

Regardless of the type of interventions taken for HAI prevention, the inclusion of monetary valuations for morbidity and mortality risk reductions in cost-benefit analyses of HAI disease prevention potentially provide economic justification for interventions that might not otherwise be considered cost saving, as illustrated by this analysis. Our model shows that the total net benefits from having AS programs and enhanced infection control are significantly large enough to cover the total investment costs in AS programs (as opposed to just 25% in our model) as the total annualized intervention cost (totaling $14.6 billion for 2015–2020 at a 3% discount rate) is only $2.7 billion.

Along with the requirement to include AS programs, CMS also proposed additional changes to the conditions of participation including a requirement that hospitals, (1) identify a qualified infection preventionist or infection control professional as an officer responsible for their infection control program and (2) conduct a review of their infection control program [26]. Other changes affecting the writing of restraint and seclusion orders for violent/self-destructive patients by a licensed practitioner, the granting of dietary ordering privileges to qualified dieticians or nutritionist professionals (in critical access hospitals), and the implementation of quality assessment and improvement programs were also proposed. While lacking data on the potential cost savings associated with many of these changes and ignoring the economic value of morbidity and mortality risk reductions from averted infections, the accompanying regulatory impact analysis concluded that the overall proposal resulted in a net benefit to society of $284 million with the majority of costs coming from changes affecting infection control programs and the addition of AS programs. Regardless of the differences in the calculated cost of the proposed CMS rule and the cost of our CDI prevention program, the additional economic benefits of morbidity risk reductions alone can be readily applied to the CMS analysis and would significantly increase the magnitude of net benefits.

A potential limitation to VSL studies of HAI prevention is the variability in methodology and quality of studies that generate attributable mortality estimates given the range of published estimates currently available [33]. The dominance of VSL estimates in the calculation of benefits highlights the need for attributable mortality measures that accurately and consistently reflect the mortality impacts of HAIs. Additional research is needed to improve the measurement of attributable mortality associated not only with HAI but with any cause of disease-related death. Given the decades of research on the VSL, the measurement of the VSL and the estimates currently used are generally accepted for injury-related risk reduction while the evidence suggests that VSL estimates for illness-related risks are probably similar [59]. However, the quality of data on mortality associated with disease should also be scrutinized as the new HHS guidelines do not directly address this issue. Use of unadjusted-crude mortality estimates, as opposed to age-adjusted or risk-adjusted estimates can produce significantly different estimates of the benefits of mortality risk reductions in cases of HAI [55, 60].

In the case of CDI disease, an important factor that likely increased attributable mortality from 2000 through 2010 was the emergence and spread of the epidemic, hyper-virulent, North American Pulse-field type 1 (NAP1) or ribotype 027 strain [61, 62]. Although declines of the NAP1/027 strain in the United States have not been as dramatic as that seen in places like England, where declines have occurred, it is likely that declines in attributable mortality have followed [58, 63]. Ironically, however, it may be AS, alone or in combination with infection control, that has led to most dramatic declines in NAP1/027 [64]. In addition, the development of new therapies and recommendations for generally more aggressive treatment of HAIs, like CDI, may also result in declines in attributable mortality but also add their own costs that must be considered [64, 65]. However, in the case of AS there may be additional impacts on morbidity and mortality that are yet to be fully understood as growing evidence suggests that the effect of unnecessary antibiotics on the microbiome may result in other adverse outcomes among hospitalized patients such as sepsis [66]. Such impacts, even if only partially realized, could even more dramatically sway the cost benefits in favor of aggressive stewardship interventions.

While our analysis illustrates the potential impact on benefits measurement when the value of mortality risks are considered with regulations that impact health, this analysis also illustrates the role that cost-benefit analysis, as opposed to cost-effectiveness analysis, has in rule making that involves public health and safety by the US Federal Government. The cost-benefit analysis described here can easily be adopted by other countries as VSL estimates, especially for mortality risk reductions related to air quality improvements, have been developed for many locations, including Europe, Asia and Australia [67–69]. While the National Health Service in the United Kingdom relies on cost-effectiveness analysis in their decision-making for assessing adoption of new healthcare interventions, HM Treasury has had guidelines (referred to as “The Green Book”) for government ministries on how to incorporate the “Value of a Prevented Fatality” (another name for the VSL) in policy assessments and evaluations of government actions that involve risks to life and health [70]. To better understand the characteristics of VSL estimates from different countries, researchers at the Organization for Economic Co-operation and Development (OECD) have conducted a meta-analysis of VSL estimates related to environmental, health and transport polices (from stated-preference studies) that have been done around the globe [69, 71]. OECD has also published a VSL user’s guide to better inform policy makers on how to incorporate VSL in policy decisions [72]. The median and mean VSL values (using the full dataset) of $2.4 and $7.4 million (in 2005 US dollars), along with other evidence from this study, can potentially be adopted for use in a global economic assessments of antimicrobial resistance [69].

## Conclusion

Although progress has been made, HAIs still pose a serious threat to patients across healthcare settings. A recent study on the prevalence of HAIs in US acute care hospitals estimated that the total number of HAIs occurring annually was 722,000, of which there were 75,000 HAI-associated deaths [1, 73]. Along with CDI, carbapenem-resistant Enterobacteriaceae (CRE) has been classified as an urgent threat to the public health in the National Action Plan for Combating Antibiotic-resistant Bacteria (White House 2015) [7]. The Plan has set targets for a reduction in incidence of both CRE and CDI, calling for 60% reduction in hospital-acquired CRE and 50% in overall CDI infections by 2020.

Our study accounted for the economic value of morbidity and mortality risk reductions, components of the total societal health benefits that have not previously been included in cost-benefit analyses of HAI prevention programs. As the US federal government intensifies its efforts to control antibiotic resistant infections, our results suggest that these ambitious goals can produce very large net societal benefits. As these benefits accrue mostly to patients, policy makers can address how the burden for the additional prevention costs should be shared among patients, third party payers and healthcare providers.

## Appendix

I. Hospital discharges used to calculate intervention costs.

The number of hospital discharges used to help calculate intervention costs were from the National Inpatient Sample (NIS).[27] We fitted a linear trend line over the number of yearly discharges for 2003 To 2014 which was then used to extrapolate annual discharges for 2015 to 2020. The equation for the estimated fitted trend line was:

1 $$ \mathrm{y}=-188,575\left({\mathrm{discharges}}_{\mathrm{i}}\right)+\mathrm{40,000,000}\ \mathrm{where}\ \mathrm{i}=\left(2003,2004,.\dots, 2014\right); $$

R^2^ (coefficient of determination) = (0.51)

The extrapolated number of discharges used to calculate intervention costs per discharge for 2015–2020 was 35,170,243; 34,981,668; 34,793,093; 34,604,518; 34,415,943; and 34,227,368 respectively. To calculate the per discharge initial year costs (2015) from federal investments to promote AS (which took place between 2009 and 2014), the total costs of these investments over these years was divided by the predicted number of discharges for 2015 (35,232,942) based on the trend analysis.

II. Deriving the Monetarized Value or Willingness-to-Pay per Quality-Adjusted Life Year (QALY).

We followed HHS guidelines to calculate the willingness-to-pay (WTP) or dollars for an expected QALY. The WTP for a QALY formula (where r is the discount rate) from Hirth et al. is:

2 $$ Value\ of\ Stisitical\ Life\ (VSL)=\sum \limits_{t=0}^{50}\frac{Pop\_{QALY}_{t+50}\ast X}{{\left(1+r\right)}^t}, $$

where we have assumed the average population age is 40 and expected life expectancy (probability of survival) is calculated for the next 50 years [37]. To derive the final quality weights (Pop_QALY_t + 50_) to be used in the above formula, each additional year of life must be adjusted (multiplied) by (1) a QALY weight to reflect the decline in quality of life with age, (2) the conditional probability of survival into the next year, and (3) a population weight that is a weighted average of the QALY weights for males and females based on proportion of the population. The QALY weights associated with male (M_QALY) and females (F_QALY) age 40 to 90 are the SF-6D scores taken from Hanmer and Kaplan [35]. As our study did not have gender-stratified incidence rates for HCA-CDI, we constructed a population weighted QALY weight (Pop_QALY) that combined the QALYs for males and females based on their proportion to total population (see Table 4). The data used to calculate the probability of survival were taken from 2013 life tables for males and females in the US (actually expressed as the probability of dying between ages x to x + 1 in Table 5) [36]. Table 5 also shows the data used to calculate Pop_QALY where the SF-6D is multiplied by (1) probability of surviving to the next year (Age_QALY which is calculated by the formula 1 - probability of dying between ages x to x + 1), and (2) the population weight for male and females respectively (Pop_Wt which is the population weighted QALY for females and males (F_QALY and M_QALY). The final QALY for the population (Pop_QALY) is the addition of F_QALY and M_QALY. Once applied to formula 2, the willingness-to-pay per QALY were calculated. For example, the 2016 VSL low estimate of $4,368,932 (3% discount rate) is divided by the discounted sum of Pop_QALY. This calculation ($4,368,932/ 20.1945) results in a willingness-to-pay for a QALY of $216,343. At the 7% discount rate, the discounted sum of Pop_QALY is 11.4027. These results are presented in Table 2.

III. Deriving the Quality-Adjusted Life Days and the Value of Morbidity Risk Reductions for Mild/Moderate CDI Disease

**Table 4:** Population by Age and Sex: 2013 (Numbers in thousands, civilian noninstitutionalized population^a^)

Age	Both sexes		Male			Female		
	Number	Cell Percent	Number	Cell Percent	Row Percent	Number	Cell Percent	Row Percent
All ages	311,116	100.0	152,335	100.0	0.490	158,781	100.0	0.510
.40 to 44 years	20,657	6.6	10,162	6.7	0.492	10,495	6.6	0.508
.45 to 49 years	21,060	6.8	10,319	6.8	0.490	10,742	6.8	0.510
.50 to 54 years	22,386	7.2	10,926	7.2	0.488	11,460	7.2	0.512
.55 to 59 years	20,880	6.7	10,099	6.6	0.484	10,781	6.8	0.516
.60 to 64 years	17,611	5.7	8224	5.4	0.467	9387	5.9	0.533
.65 to 69 years	14,437	4.6	6900	4.5	0.478	7537	4.7	0.522
.70 to 74 years	10,264	3.3	4704	3.1	0.458	5561	3.5	0.542
.75 to 79 years	7598	2.4	3233	2.1	0.426	4364	2.7	0.574
.80 to 84 years	5692	1.8	2490	1.6	0.437	3202	2.0	0.563
.85 years and over	5296	1.7	1971	1.3	0.372	3325	2.1	0.628

Details may not sum to totals because of rounding

US Census Bureau, Current Population Survey, Annual Social and Economic Supplement, 2013. Internet release date: March 2016, https://www2.census.gov/programs-surveys/demo/tables/age-and-sex/2013/age-sex-composition/

^a^ Plus armed forces living off post or with their families on post

**Table 5:** Calculation of Population QALY Weights

Age (years)	Female Probability of dying between ages *x* and *x* + 1^a^	Male Probability of dying between ages *x* and *x* + 1^b^	Females				Males				Weighted Population QALY (F_QALY + M_QALY)
	*q*_*x*_	*q*_*x*_	SF-6D	Age_QALY	Pop_WT	F_QALY	SF-6D	Age_QALY	Pop_WT	M_QALY	Pop_QALY
40–41	0.001299	0.002105	0.770	0.769	0.508	0.391	0.808	0.806	0.492	0.397	0.787
41–42	0.001403	0.002253	0.770	0.769	0.508	0.391	0.808	0.806	0.492	0.397	0.787
42–43	0.001523	0.002425	0.770	0.769	0.508	0.391	0.808	0.806	0.492	0.397	0.787
43–44	0.001663	0.002631	0.770	0.769	0.508	0.391	0.808	0.806	0.492	0.396	0.787
44–45	0.001827	0.002875	0.770	0.769	0.508	0.390	0.808	0.806	0.492	0.396	0.787
45–46	0.002004	0.003143	0.770	0.768	0.510	0.392	0.808	0.805	0.490	0.395	0.787
46–47	0.002197	0.003443	0.770	0.768	0.510	0.392	0.808	0.805	0.490	0.395	0.786
47–48	0.002421	0.003798	0.770	0.768	0.510	0.392	0.808	0.805	0.490	0.394	0.786
48–49	0.002674	0.004205	0.770	0.768	0.510	0.392	0.808	0.805	0.490	0.394	0.786
49–50	0.002941	0.004645	0.770	0.768	0.510	0.392	0.808	0.804	0.490	0.394	0.786
50–51	0.003212	0.005090	0.756	0.754	0.512	0.386	0.787	0.783	0.488	0.382	0.768
51–52	0.003484	0.005541	0.756	0.753	0.512	0.386	0.787	0.783	0.488	0.382	0.768
52–53	0.003760	0.006026	0.756	0.753	0.512	0.386	0.787	0.782	0.488	0.382	0.767
53–54	0.004046	0.006565	0.756	0.753	0.512	0.385	0.787	0.782	0.488	0.382	0.767
54–55	0.004351	0.007158	0.756	0.753	0.512	0.385	0.787	0.781	0.488	0.381	0.767
55–56	0.004680	0.007794	0.756	0.752	0.516	0.389	0.787	0.781	0.484	0.378	0.766
56–57	0.005028	0.008451	0.756	0.752	0.516	0.388	0.787	0.780	0.484	0.377	0.766
57–58	0.005391	0.009124	0.756	0.752	0.516	0.388	0.787	0.780	0.484	0.377	0.765
58–59	0.005766	0.009803	0.756	0.752	0.516	0.388	0.787	0.779	0.484	0.377	0.765
59–60	0.006166	0.010500	0.756	0.751	0.516	0.388	0.787	0.779	0.484	0.377	0.765
60–61	0.006598	0.011256	0.756	0.751	0.533	0.400	0.781	0.772	0.467	0.361	0.761
61–62	0.007083	0.012076	0.756	0.751	0.533	0.400	0.781	0.772	0.467	0.360	0.760
62–63	0.007638	0.012921	0.756	0.750	0.533	0.400	0.781	0.771	0.467	0.360	0.760
63–64	0.008279	0.013773	0.756	0.750	0.533	0.400	0.781	0.770	0.467	0.360	0.759
64–65	0.009003	0.014646	0.756	0.749	0.533	0.399	0.781	0.770	0.467	0.359	0.759
65–66	0.009813	0.015569	0.756	0.749	0.522	0.391	0.781	0.769	0.478	0.367	0.758
66–67	0.010703	0.016603	0.756	0.748	0.522	0.390	0.781	0.768	0.478	0.367	0.758
67–68	0.011675	0.017800	0.756	0.747	0.522	0.390	0.781	0.767	0.478	0.367	0.757
68–69	0.012753	0.019228	0.756	0.746	0.522	0.390	0.781	0.766	0.478	0.366	0.756
69–70	0.013958	0.020906	0.756	0.745	0.522	0.389	0.781	0.765	0.478	0.365	0.755
70–71	0.015325	0.022826	0.738	0.727	0.542	0.394	0.757	0.740	0.458	0.339	0.733
71–72	0.016892	0.024998	0.738	0.726	0.542	0.393	0.757	0.738	0.458	0.338	0.731
72–73	0.018650	0.027356	0.738	0.724	0.542	0.392	0.757	0.736	0.458	0.337	0.730
73–74	0.020487	0.029913	0.738	0.723	0.542	0.392	0.757	0.734	0.458	0.337	0.728
74–75	0.022554	0.032679	0.738	0.721	0.542	0.391	0.757	0.732	0.458	0.336	0.726
75–76	0.024831	0.035524	0.738	0.720	0.574	0.413	0.757	0.730	0.426	0.311	0.724
76–77	0.027514	0.039010	0.738	0.718	0.574	0.412	0.757	0.727	0.426	0.310	0.722
77–78	0.030684	0.043116	0.738	0.715	0.574	0.411	0.757	0.724	0.426	0.308	0.719
78–79	0.034250	0.047647	0.738	0.713	0.574	0.409	0.757	0.721	0.426	0.307	0.716
79–80	0.038265	0.052626	0.738	0.710	0.574	0.408	0.757	0.717	0.426	0.305	0.713
80–81	0.042554	0.058301	0.698	0.668	0.563	0.376	0.725	0.683	0.437	0.299	0.675
81–82	0.047066	0.064637	0.698	0.665	0.563	0.374	0.725	0.678	0.437	0.297	0.671
82–83	0.052561	0.071412	0.698	0.661	0.563	0.372	0.725	0.673	0.437	0.295	0.667
83–84	0.058864	0.079031	0.698	0.657	0.563	0.370	0.725	0.668	0.437	0.292	0.662
84–85	0.066285	0.087905	0.698	0.652	0.563	0.367	0.725	0.661	0.437	0.289	0.656
85–86	0.074167	0.098958	0.698	0.646	0.628	0.406	0.725	0.653	0.372	0.243	0.649
86–87	0.083469	0.110149	0.698	0.640	0.628	0.402	0.725	0.645	0.372	0.240	0.642
87–88	0.093753	0.122333	0.698	0.633	0.628	0.397	0.725	0.636	0.372	0.237	0.634
88–89	0.105076	0.135536	0.698	0.625	0.628	0.392	0.725	0.627	0.372	0.233	0.625
89–90	0.117487	0.149773	0.698	0.616	0.628	0.387	0.725	0.616	0.372	0.229	0.616

^a,b^ NCHS, National Vital Statistics System, Mortality

www.cdc.gov/nchs/data/nvsr/nvsr66/nvsr66_03.pdf

As we lack the incidence data to predict the cases of severe HCA-CDI, we have assumed that, at a minimum, all cases are at least mild or moderate. To estimate the value of morbidity risk reductions for mild/moderate HCA-CDI disease, we selected published community preference-based QALY weights (EQ-5D scores) to represent the decline in quality of life due to a case of HCA-CDI and also recurrent disease [38]. Lacking a QALY weight specifically for HCA-CDI disease, the surrogate measures used included (1) 0.80 - the unadjusted EQ-5D score for the 25% percentile for the general sample from the Medical Panel Expenditure Study (MEPS) to represent the baseline quality of life for patients that could get an HCA-CDI infection, and (2) 0.704 – the unadjusted EQ-5D for the 25th percentile for MEPS respondent reported having other gastrointestinal disorders (Chronic Disease Classication 155). As the length of hospital stay for either a case or recurrence of CDI is on average 9.5 days and 8.8 days respectively, these QALYs must be adjusted down due to the acute nature of HCA-CDI and recurrent CDI disease. Eqs. 3 and 4 were used to make these adjustments

3 $$ Lost\ QALYs\ from\  HCA- CDI\  Disease=\left[1-\left( EQ-5{D}_{case}\right)\right]\ast \left(\frac{Length\ of\ Hospital\ Stay}{365}\right) $$

4 $$ Lost\ QALYs\ from\ Recurrent\ Disease=\left[1-\left( EQ-5{D}_{recurrent}\right)\right]\ast \left(\frac{Length\ of\ Hospital\ Stay}{365}\right) $$

The calculations for HCA-CDI and recurrent cases are as follows:

HCA-CDI: 0.005205479 = (1–0.80)*(9.5/365)

Recurrent: 0.00704 = (1–0.708)*(8.8/365)

The results are used to adjust the willingness-to-pay for QALY to provide the monetary estimate of the value of reducing the risk of a mild/moderate case of HCA-CDI. For example, the willingness-to-pay for a QALY for HCA-CDI using the 2016 low VSL estimate (3% discount rate) of $216,343 results in a lost QALY estimate of $1126 ($216,343 * 0.005205479). The final calculations for these values are also in Table 2.

IV. Sensitivity Analysis Using Quality-Adjusted Life Days and the Value of Morbidity Risk Reductions From Mild/Moderate CDI Disease Based on Ages 65 to 90

Recognizing that the age distribution of patients with HCA-CDI is older than for the general population, we recalculated the value of morbidity risk reductions based on a population age range of 65 to 90. From Table 5, the revised discounted years of life gained (Pop_QALY) to be used in the calculations are now 13.1215 (3% discount rate) and 9.1220 (7% discount rate). The revised values for mortality and morbidity risk reductions are in Table 6. The net benefit calculations based on the age-adjusted VSL or value per QALY estimates are presented in Table 7.

**Table 6:** Model Inputs: Cases and Deaths Averted; Value Per QALY, Value per QALD 2015–2020 (2015 $) for Ages 65 to 90 Years Old

	7% discount rate 10% effectiveness	3% discount rate 10% effectiveness	7% discount rate 50% effectiveness	3% discount rate, 50% effectiveness		
Cases Averted^a^						
Inpatient Cases Averted						
HCA-CDI	167,699	184,749	838,493	923,743		
Recurrent	35,892	39,555	179,460	197,775		
Total	203,591	224,304	1,017,953	1,121,518		
Deaths Averted						
35% Attributable Mortality	5625	6199	28,123	30,996		
50% Attributable Mortality	8035	8856	40,176	44,280		
VSL Estimates (2015 $)^b^						
3% DR	2015	2016	2017	2018	2019	2020
Low	$4,500,000	$4,368,932	$4,335,941	$4,301,166	$4,175,889	$4,140,522
Central	$9,400,000	$9,320,388	$9,143,180	$8,968,388	$8,884,870	$8,712,349
High	$14,400,000	$14,174,757	$13,950,419	$13,727,125	$13,505,003	$13,370,436
7% DR	2015	2016	2017	2018	2019	2020
Low	$4,500,000	$4,236,288	$3,959,148	$3,782,363	$3,611,765	$3,375,481
Central	$9,400,000	$9,037,415	$8,534,163	$8,140,304	$7,684,607	$7,253,694
High	$14,400,000	$13,744,402	$13,021,197	$12,333,794	$11,680,602	$11,060,088
Value Per QALY (2015 $)						
3% DR	2015	2016	2017	2018	2019	2020
Low	$342,948	$332,959	$330,445	$327,795	$318,248	$315,552
Central	$716,381	$710,313	$696,808	$683,487	$677,122	$663,974
High	$1,097,434	$1,080,268	$1,063,171	$1,046,154	$1,029,226	$1,018,971
7% DR	2015	2016	2017	2018	2019	2020
Low	$493,311	$461,039	$440,452	$420,586	$393,071	$375,173
Central	$1,030,473	$983,549	$928,780	$876,967	$836,322	$789,426
High	$1,578,597	$1,495,815	$1,417,108	$1,342,297	$1,271,210	$1,211,495
Value Per QALD (2015 $)						
3% DR - Case	2015	2016	2017	2018	2019	2020
Low	$1785	$1733	$1720	$1706	$1657	$1643
Central	$3729	$3698	$3627	$3558	$3525	$3456
High	$5713	$5623	$5534	$5446	$5358	$5304
3% DR - Recurrent	2015	2016	2017	2018	2019	2020
Low	$2414	$2344	$2326	$2308	$2240	$2221
Central	$5043	$5001	$4906	$4812	$4767	$4674
High	$7726	$7605	$7485	$7365	$7246	$7174
7% DR - Cases	2015	2016	2017	2018	2019	2020
Low	$2568	$2400	$2293	$2189	$2046	$1953
Central	$5364	$5120	$4835	$4565	$4353	$4109
High	$8217	$7786	$7377	$6987	$6617	$6306
7% DR - Recurrent	2015	2016	2017	2018	2019	2020
Low	$3473	$3246	$3101	$2961	$2767	$2641
Central	$7255	$6924	$6539	$6174	$5888	$5558
High	$11,113	$10,531	$9976	$9450	$8949	$8529

*VSL* value of statistical life, *QALY* quality-adjusted life year, *QALD* quality-adjusted life day, *DR* discount rate

^a^To calculate the number of cases, we took national incidence rates from Lessa et al. (2015) and applied them to projections of the US population (by year of age) for 2015–2020 (United States Census Bureau 2016) [24, 28]. Rates of attributable CDI mortality were derived by the authors’ based on analysis by Kwon et al. (2015) [29].

^b^The base estimates for the VSL were taken from the new HHS guidelines for conducting regulatory impact analysis (US Department of Health and Human Services 2017) [21].

**Table 7:** Benefits and Costs of a Comprehensive CDI Prevention Program 2015–2020 (2015 $) Using Age Adjusted VSL Estimates

	7% discount rate, 10% effectiveness	3% discount rate, 10% effectiveness	7% discount rate, 50% effectiveness	3% discount rate, 50% effectiveness
Total Intervention Costs (in billions)	$3.7	$4.7	$3.3	$4.2
Total Benefits (in billions)				
Direct Medical Cost Savings				
Savings in Patient Care Costs	$1.5	$1.8	$7.6	$9.1
Savings in Expenditures for Antibiotics	$3.8	$4.2	$3.8	$4.2
Benefits of Morbidity Risk Reduction				
Low $/Lost QALY from HCA-CDI	$0.5	$0.4	$2.6	$2.0
Central $/ Lost QALY from HCA-CDI	$1.0	$0.9	$5.1	$4.3
High $/ Lost QALY from HCA-CDI	$1.6	$1.3	$7.8	$6.5
Benefits of Mortality Risk Reduction				
Low VSL				
35% Attributable Mortality Proportion	$2.4	$2.0	$12.2	$10.1
50% Attributable Mortality Proportion	$3.5	$2.9	$17.3	$14.5
Central VSL				
35% Attributable Mortality Proportion	$5.1	$4.3	$25.5	$21.4
50% Attributable Mortality Proportion	$7.3	$6.1	$35.0	$30.6
High VSL				
35% Attributable Mortality Proportion	$7.8	$6.5	$39.0	$32.7
50% Attributable Mortality Proportion	$11.3	$9.3	$55.7	$46.7
Range of Total Net Benefits				
Low	$4.5 - $5.6	$3.7 - $4.6	$22.9 - $28.0	$21.2 - $25.6
Central	$7.7 - $9.9	$6.5 - $8.3	$38.7 - $48.2	$34.8 - $44.0
High	$11.0 - $14.5	$9.1 - $11.9	$54.9 - $71.6	$48.3 - $62.3

*HCA-CDI* healthcare-associated *Clostridioides difficile* infection, *VSL* value of a statistical life, *QALY* quality-adjusted life year

## Abbreviations

AS: Antibiotic stewardship
AU: Antibiotic utilization option of the antimicrobial use and resistance module of the national healthcare safety network
CDI: *Clostridioides difficile* infection
CMS: Centers for medicare and medicaid services
CPI-U: Consumer price index for urban consumers
HAI: Healthcare-associated infection
HCA-CDI: Healthcare-associated *Clostridioides difficile* infection
HHS: Department of Health and Human Services
HRQL: Health-related quality of life
OMB: Office of management and budget
QALD: Quality-adjusted life day
QALY: Quality-adjusted life year
US: United States
VSL: Value of statistical life
